# Ultrasensitive and Self-Powered Multiparameter Pressure–Temperature–Humidity Sensor Based on Ultra-Flexible Conductive Silica Aerogel

**DOI:** 10.3390/gels9020162

**Published:** 2023-02-17

**Authors:** Song He, Chunhua Du, Hongliang Sheng, Chunxiang He, Xinyu Liu, Xin Jin, Qilin Chen, Fuliang Tian

**Affiliations:** 1 School of Safety Science and Emergency Management, Wuhan University of Technology, Luoshi Road 122, Wuhan 430070, China; 2 Wuhan Building Material Industry Design & Research Institute Co., Ltd., Wuhan 430200, China

**Keywords:** ultrasensitive, self-powered, multiparameter sensing, Seebeck effect, silica aerogel

## Abstract

The application of silica aerogel has been limited because of its poor mechanical properties. In order to expand the application scope of silica aerogel, this study fabricated an ultra-flexible conductive silica aerogel as a multiparameter sensor. The sample is fabricated by introducing poly (3,4-ethylenedioxythiophene):polystyrene sulfonate (PEDOT:PSS) on a base of ultra-flexible silica aerogel, which was prepared by a diene synthesis reaction at atmospheric pressure. The pressure, temperature, and humidity can be converted into electrical signals. The pressure sensitivity can reach up to 54.88 kPa^−1^, and the detection limit is as low as 5 Pa. The temperature resolution is up to 0.1 K, and the response time of humidity is within 4 s. More importantly, the developed multiparameter sensor can be self-powered to realize multiparameter sensing of pressure, temperature, and humidity. The ultra-flexible conductive silica aerogel is a promising candidate for monitoring human activities and fire-affected areas.

## 1. Introduction

In recent years, silica aerogel has attracted much attention because of its unique properties, including ultra-low density [1], high specific surface area [2,3], and low thermal conductivity [4]. As a result, silica aerogel possesses various potential technical applications [5], for instance in thermal insulation, the aerospace industry, carrier media for sophisticated catalysts, national defense, military, etc. However, silica aerogel suffers from poor mechanical properties [6,7,8], restricting its application. In addition, the application of silica aerogel in various fields mainly depends on the characteristics of high porosity, hydrophobicity, and low thermal conductivity [9,10]. Silica aerogel has not yet obtained the function of electric conduction, and there are few reports on the application of electrical conduction involving silica aerogel. The conductive aerogel most often studied previously is graphene aerogel [11,12] with high costs and complicated manufacturing processes [13].

With the development of artificial intelligence technology, multiparameter sensors, as an important part of smart components, have attracted more and more scholars’ attention. At present, the research on multiparameter sensors is mainly concentrated in the array sensor [14,15], integrated sensor [16], and wireless multiparameter sensor network [17]. Array sensors are usually composed of several specific function sensors. Various sensors sensing a certain stimulus are based on different sensing materials. Each specific functional sensor responds to a specific stimulus and then generates a readable signal. Therefore, several signals can be detected at the same time without any crosstalk. The sensor arrays usually require many processes, including a large number of materials and special production steps, which will lead to high costs. Different sensors and matrix arrays are combined with several Si chips, so a lot of separate connecting devices are required. This will also generate a lot of waste, which limits their large-scale application. At the same time, the research on the application of multifunctional materials in the sensors is mainly concentrated on electrochemical sensors and biosensors, and the research on physical parameter sensors is mostly limited to single-parameter sensors. Therefore, it is still a challenge to realize multiparameter sensing by one sensor.

The conductivity of conductive polymers depends on many factors, such as temperature, humidity, dopants, etc. Therefore, conductive polymers have potential as inductive materials for multiparameter sensors. For example, polymer poly (3,4-ethylenedioxythiophene): polystyrene sulfonate (PEDOT:PSS) is the most notable example of a conductive polymer. Polystyrene sulfonate (PSS) is added to the conductive polymer poly (3,4-ethylenedioxythiophene) (PEDOT), which can make PEDOT uniformly dispersed in the solution [18].

We have previously reported on the ultra-flexible silica aerogel, which is fabricated by a diene synthesis reaction [19]. The aerogel was endowed with excellent conductivity by introducing PEDOT:PSS and applied to the field of multiparameter sensors. The PEDOT:PSS used in this study provides the electronic thermal voltage and the ionic thermal voltage. The ultra-flexible conductive silica aerogel forms the mechanical structure of the sensors. The ultra-flexible conductive silica aerogel has decoupling sensitivity to applied pressure, range of temperature, and relative humidity. When the aerogel is placed in a non-power circuit, the applied pressure can be read as electric resistance, the range of temperature can be obtained by the thermal voltage, and the relative humidity would be worked out from the thermal voltage peak. The sensing process can be accomplished without an additional power supply for the intrinsic thermoelectric mechanism and humidity-sensing mechanism. The self-powered multiparameter sensor can measure several parameters at the same time without any major crosstalk.

## 2. Results and Discussion

### 2.1. Compositional Analysis

**Figure 1:**
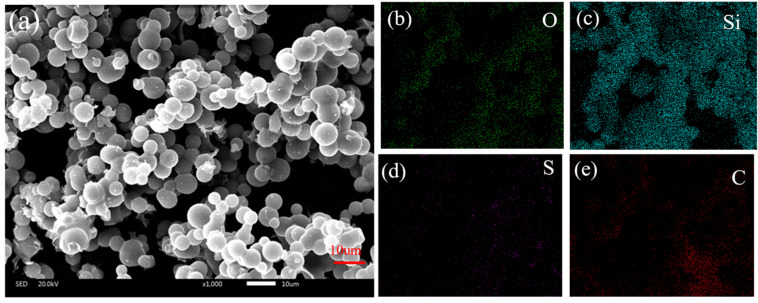
(**a**) Scanning electron microscopy of silica aerogel with PEDOT:PSS; (**b**–**e**) elemental mapping of silica aerogel with PEDOT:PSS.

The microstructure and content elements of aerogel without PEDOT:PSS and aerogel with PEDOT:PSS were observed by SEM-EDX, as shown in Figure 1. The elemental composition (Si, 24.21%, C, 53.28%, O, 21.27%, and S, 0.81%) of the conductive aerogel was different from that of silica aerogel without PEDOT:PSS. Compared to the silica aerogel without PEDOT:PSS (Figure S2), the S element indicates that the PEDOT:PSS has been successfully introduced onto ultra-flexible silica aerogel (Table 1). The conductive material PEDOT:PSS on the surface of the porous structure endowed the aerogel with excellent conductivity, which lays the foundation for a multiparameter pressure–temperature–humidity sensor.

### 2.2. Experimental Methodology

A magnetic stirrer was used to stir at an adjustable frequency. A constant temperature oven that provided a temperature of 40–80 °C, was used for gel aging and drying of the aerogel. Ammeters and voltmeters are measuring devices that measure the changes in sensor electrical signals caused by pressure, temperature, and humidity.

### 2.3. Pressure Sensing

This study investigated the pressure sensing of a multiparameter sensor. The mechanical properties of ultra-flexible conductive silica aerogel determined the performance of the pressure sensor (Figure S3). Mechanical properties are an important factor to be considered in the practical application of sensors. The sensing of pressure parameters is mainly attributed to the piezoresistive effect [20,21]. The piezoresistive effect refers to the change in resistivity caused by applied pressure. For most devices, the change in resistivity caused by pressure is mainly due to the geometric dimension. When there are no pressure and temperature changes, the resistance of conductive aerogel is constant, and the sample provides no thermal voltage. Therefore, the current through the sample has a linear response to the applied voltage, and the current is zero when the voltage is zero. When the pressure is applied to the multiparameter sensor, the changes in force cause changes in ultra-flexible aerogel in thickness and volume, which causes a change in the conductive path of the porous structure (Figure S4). The resistance of the sensor will be changed by applied pressure.

**Figure 2:**
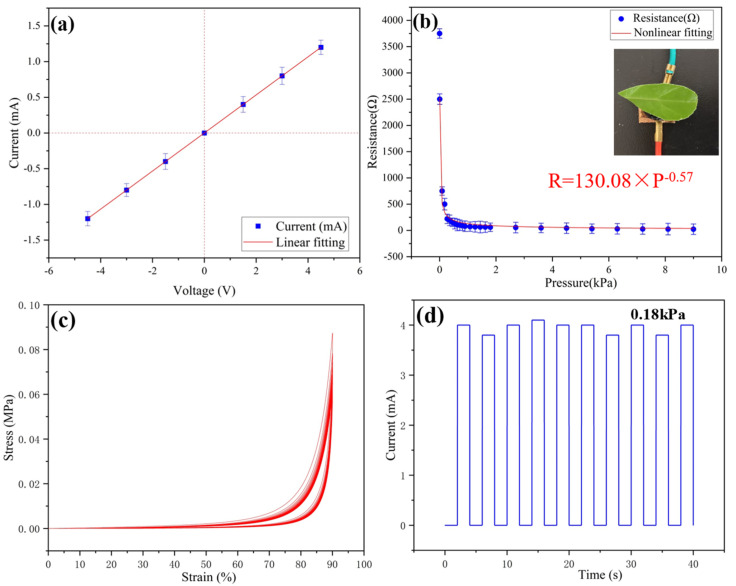
(**a**) Variations in sensor current with voltage without applied pressure; (**b**) resistance of the sensor with different applied pressures; (**c**) stress-strain curves of ultra-flexible silica aerogel for 20 cycles; (**d**) stability test of the conductive aerogel sensing property at 0.18 kPa.

As shown in Figure 2a, different pressures correspond to different resistances of the sensor. The resistance value can be calculated by the slope of the I-V curves. Therefore, the corresponding pressure can be obtained by recording the current value under a certain voltage. As shown in Figure 2b, the electric resistance of the sensor changes with the applied pressure, and the resistance changes from 3750 Ω (without pressure) to 23 Ω (at 9 kPa). Since the elastic modulus of the conductive gel is as low as 0.05 MPa, this gives the sample excellent flexibility. Therefore, the detection limit of the conductive aerogel is as low as 5 Pa, which means that the pressure from a leaf can be detected. As shown in Figure 2c, no permanent deformation is found in samples after several cycles, revealing the excellent cyclic compressive stability of the ultra-flexible silica aerogel. As shown in Figure 2d, the load of 0.18 kPa is repeatedly pressurized and unloaded for 10 cycles, and the change in current value is recorded. It can be seen that the output current of the sensor device is relatively stable, which ensures the reliability of the sensor in long-term use.

**Figure 3:**
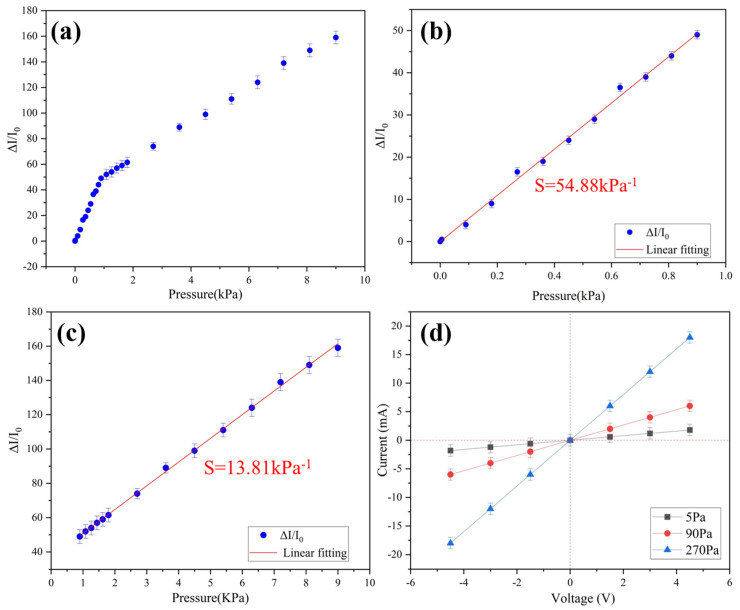
(**a**) Relative changes in current corresponding to different applied pressures; (**b**) sensitivity in the range of 0–0.9 kPa; (**c**) sensitivity in the range of 0.9–9 kPa; (**d**) I-V curves under different pressures.

In order to evaluate the pressure sensing performance of the multiparameter sensor, it is very important to evaluate several key parameters of the sensor, such as pressure, resistance change, and sensitivity. The pressure sensitivity of the device is defined as Equation (1):

S = (ΔI − I_0_)/ΔP (1)

where I_0_ is the initial current of the sensor, ΔI is the change in current caused by applied pressure, and ΔP is the change in applied pressure. The porous structure of the conductive aerogel is important to the high sensitivity and sensing range of the pressure sensing. The stress is concentrated around the pore structure, which will lead to the deformation of pores, and the contact area between pores will also change [22], which means that the conductive resistance decreases. The excellent mechanical properties of the ultra-flexible aerogel endow the pressure sensor with ultra-sensitivity. The pressure sensitivity is as high as 54.88 kPa^−1^ in the range of 0–0.9 kPa and 13.81 kPa^−1^ over 0.9 kPa (Figure 3). Compared with the previously reported pressure sensors, this sensitivity of the sample has excellent performance (Table 2).

To verify the change in electrical resistance caused by applied pressure, the conductive aerogel was connected to a small bulb by wire conductors. When the pressure on the conductive aerogel was zero, the small bulb could not emit bright light, because the electrical resistance of the aerogel is too high to supply the current to illuminate brightly. The small bulb gave out light when pressure was applied to the conductive aerogel (Video S1).

### 2.4. Temperature Sensing

**Figure 4:**
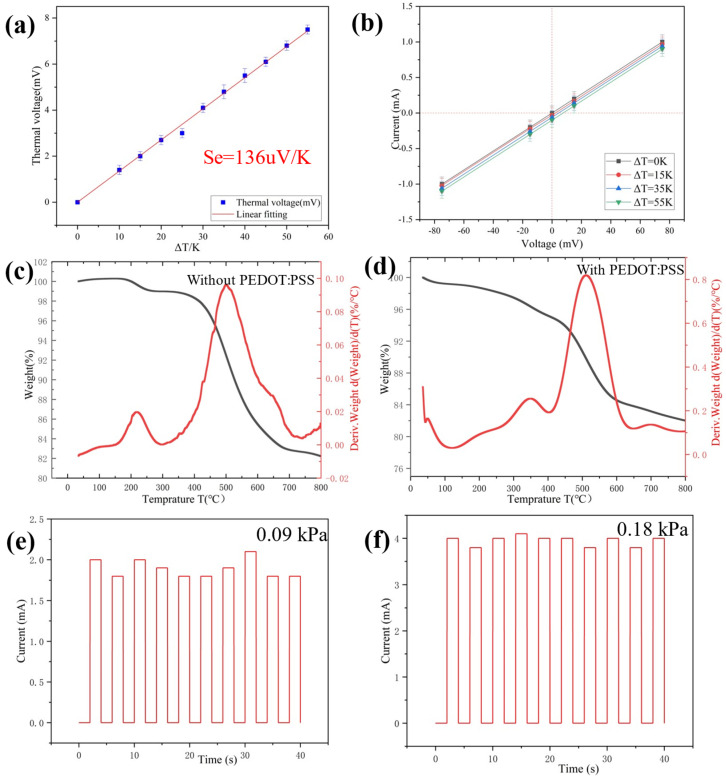
(**a**) Thermal voltage change with different temperature gradients; (**b**) I-V curves of the multiparameter sensor device under various temperature gradients. (**c**,**d**) TG-DTG curves of silica aerogel without PEDOT:PSS and with PEDOT:PSS; (**e**,**f**) Test of repeatability of 10 cycles for the pressure sensor after baking at 100 °C for 30 min.

When exerting a difference of temperature between the two sides of the sensor, a thermal voltage will be generated [33]. It is known as the electronic Seebeck effect [34,35]. The linear change of temperature will lead to the linear change of thermoelectric voltage. (Figure 4a). According to the thermoelectric mechanism, the thermal voltage (Ve) at both ends of the sensor can be calculated by Equation (2) [36]:

Ve = Se × ΔT(2)

where ΔT is the range of temperature and Se is the Seebeck coefficient. When one side of the multiparameter sensor comes into contact with objects with different temperatures, the difference in temperature between the object and the sensor can be measured by the electronic Seebeck effect. Notably, even a small ΔT of 0.1 K was clearly detected with a thermal voltage of 13.6 μV, indicating accurate temperature sensing and high resolution. As shown in Figure 4b, the effect of temperature stimulation on the change of material resistance is negligible [37,38]. Therefore, the values of temperature and pressure can be calculated by using voltage and current respectively.

The thermal behavior of ultra-flexible silica aerogel without PEDOT:PSS and conductive silica aerogel were studied by TG-DTG at a heating rate of 10 °C min^−1^ in nitrogen. The TGA-DTG plots of the aerogel are shown in Figure 4c. Between 100–250 °C, the weight loss of two samples is basically determined by the evaporation of the remaining water [39]. In the DTG profiles of both samples, the samples show an exothermic peak at 500 °C. This is caused by the oxidation of silico-methyl (Si-CH3) on the surface of the aerogel to silyl hydroxyl (Si-OH). The samples began to lose weight at 700 °C due to the condensation reaction of silyl hydroxyl (Si-OH), which leads to the loss of quality. According to Figure 4d, the initial temperature of PEDOT:PSS decomposition is about 250 °C [40]. At 800 °C, the amount of residue in the nitrogen atmosphere is more than 76%, suggesting that the conductive aerogel contains around 24% organic material. The conductive silica aerogel was thermally stable up to a temperature of 250 °C in the nitrogen atmosphere, according to the TG-DTG profiles.

According to the TG-DTG profiles, the thermal stability of the conductive silica aerogel reached about 250 °C. Furthermore, the sensor revealed stable and repeatable responses after baking at 100 °C for 30 min (Figure 4e,f).

### 2.5. Humidity Sensing

**Figure 5:**
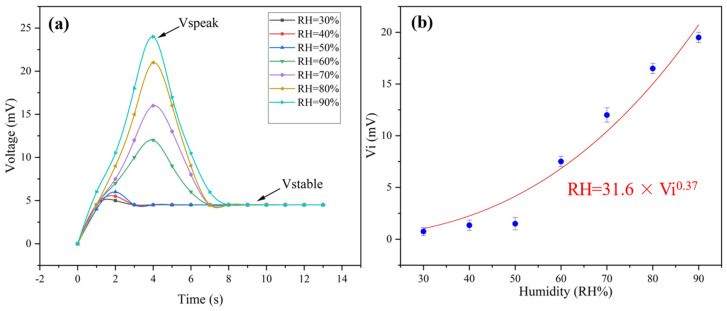
(**a**) The change in voltage with time under different humidities at ΔT = 35 K; (**b**) the change in ionic thermal voltage with different humidity gradients.

Under humid conditions, the ionization of PSS makes PEDOT:PSS ionic conductive, laying the foundation of humidity sensing. At humid conditions, the generated thermal voltage of the multiparameter sensor includes Ve and ionic contribution. The ionic thermal voltage was named Vi. As shown in Figure 5, Vi goes through a totally different variation tendency from that of Ve, which presents a power function relationship with relative humidity (Figure S5). What should be emphasized is that the peak value of the voltage (Vpeak) increases when the humidity rises. The voltage becomes constant just several seconds later (Figure 5a). As a result, the ionic thermal voltage can be calculated by Equation (3):

Vi = Vpeak − Vstable(3)

where Vpeak is the peak voltage and Vstable is the constant voltage. To our knowledge, the response time is one of the most prominent values reported for humidity sensors (Table 3).

### 2.6. Self-Powered Multiparameter Sensing Analysis

The construction of self-powered sensing devices represents an important step toward the application of sustainable sensors. As mentioned above, the surface temperature and humidity can be sensed by a conductive silica aerogel without an additional power supply for the intrinsic thermoelectric mechanism and humidity-sensing mechanism.

In a typical sensing scenario, the voltage–time curve could reflect two stages: reversed-V variation (which lasts for several seconds) and followed by constant voltage, just as shown in Figure 5a. As mentioned above, the reversed-V variation is caused by the humidity. On the other hand, the stable voltage following the reversed-V variation results from the temperature difference between the two ends of the sample.

According to Figure 5b, the relationship between humidity and voltage can be fitted as follows in Equation (4):

RH = 31.6 × Vi^0.37^ (4)

where Vi is the ionic thermal voltage value and RH is the humidity value.

The relationship between temperature and voltage is as follows in Equation (5):

ΔT = Vstable/Se(5)

where Ve is the constant value for t > 8 s, Se is the electron Seebeck constant.

The relationship between pressure and resistance is as follows in Equation (6):

R = 130.08 × P^−0.57^ (6)

where R is the resistance of conductive aerogel under pressure, P is the applied pressure.

The self-powered multiparameter sensor realized the separate measurement of pressure, temperature, and humidity by recording the voltage and current in time. The humidity is calculated by using Equation (4); after a period of time, the stable voltage can be measured. The temperature is obtained by using the relationship between temperature and voltage. After the stable current is obtained, the resistance is calculated by using the relationship between voltage and current, and the pressure is calculated according to the relationship between resistance and pressure.

## 3. Detecting Capability of the Self-Powered Multiparameter Sensor

### 3.1. Human Activity Monitoring

**Figure 6:**
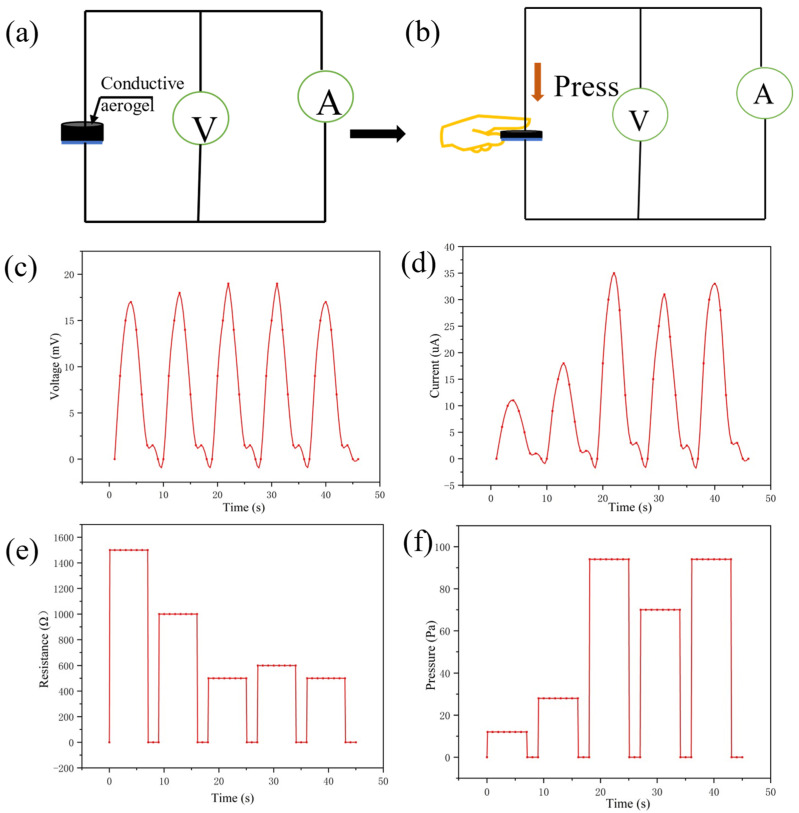
(**a**) Test circuit of the self-powered sensor; (**b**) the index finger pressed the conductive aerogel with different strengths; (**c**,**d**) changes in voltage and current during pressing; (**e**,**f**) changes in electric resistance and applied pressure during pressing.

From the above simulative experiments, the sensitivity of the sensor has excellent performance in multiple stimulation conditions. Next, as shown in Figure 6a, the conductive silica aerogel sensor was pressed by the index finger under the air humidity to detect its multiparameter sensing ability under different applied pressures. All tests do not provide voltage and rely on the thermoelectric potential provided by temperature and humidity sensing. The appearance and thickness of the aerogel would change after being pressed by the finger. Figure 6b shows that the sensor was used to record the changes in the signal under the finger’s pressure and temperature. It can respond rapidly to different pressing forces. From the voltmeter readings (Figure 6c), the temperature of the index finger and the humidity of the air can be detected. Different pressing forces can get different ammeter readings (Figure 6d).

Under indoor conditions, the multiparameter sensor showed repeatable current changes and thermal voltage responses to the pressure and temperature of the index finger. The sensor can recognize the slight pressure of the index finger contacting the conductive aerogel. The monitoring temperature remains unchanged due to the constant finger temperature. The humidity is about 80%, as concluded from Equation (4). From Equation (5) it can be inferred that the temperature of the index finger is about 35 °C. The ratio of voltage to current reflects the pressing force of the index finger. The electric resistance and the applied pressure during pressing can be seen in Figure 6e,f.

### 3.2. Fire Scene Monitoring

**Figure 7:**
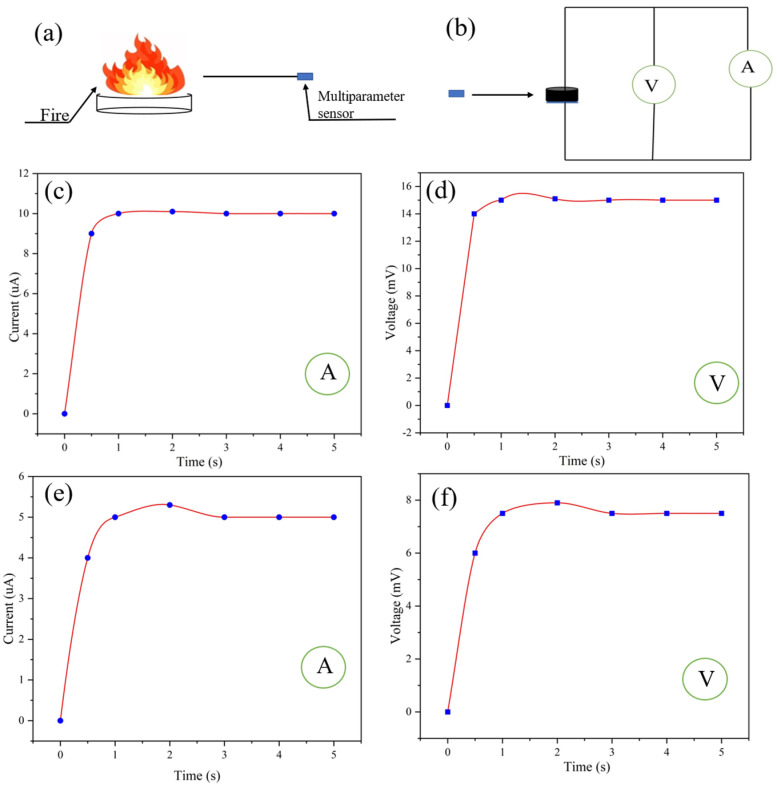
(**a**,**b**) Fire sensing at a certain distance from the fire source by the multiparameter sensor; (**c**,**d**) ammeter and voltmeter readings of the sensor 10 cm away from the fire source; (**e**,**f**) ammeter and voltmeter readings of the sensor 30 cm away from the fire source.

Because the ultra-flexible conductive silica aerogel is resistant to high temperatures, it can be used for monitoring fire-affected areas (Figure S6) and has great potential in fire safety protection. As shown in Figure 7a,b, the self-powered multiparameter sensor was used to monitor a fire at a certain distance. According to the ammeter and voltmeter readings of the sensor (Figure 7c,d), the temperature monitored at 10 cm away from the fire source is calculated to be 110 °C. According to Equation (4), the humidity at 10 cm away from the fire source is calculated to be 10%. The temperature at 30 cm away from the fire source can be obtained from the readings of the voltmeter and ammeter in the multiparameter sensor (Figure 7e,f). According to Equation (5), the humidity at 10 cm away from the fire source is calculated to be 20%. According to Equation (5), the temperature monitored at 30 cm away from the fire source is calculated to be 55 °C.

**Figure 8:**
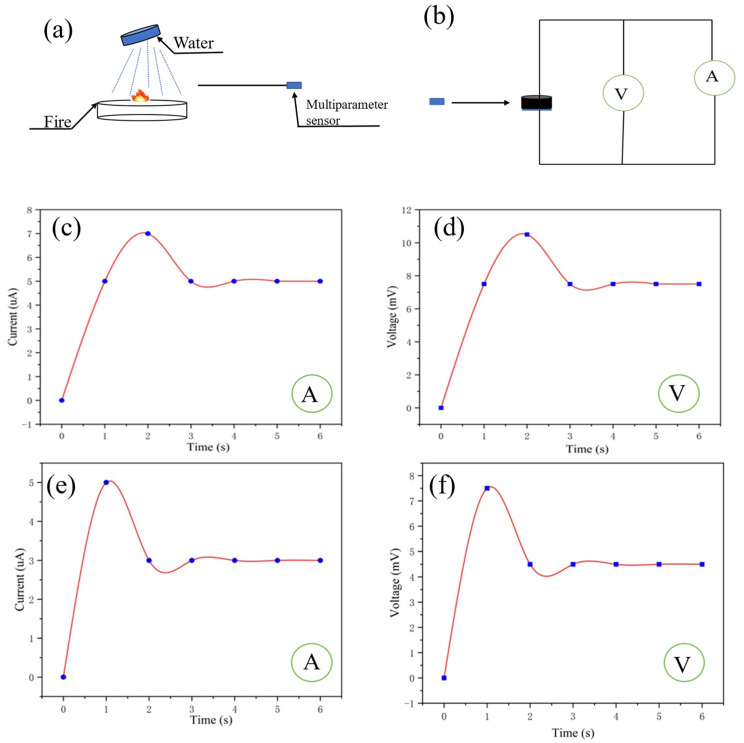
(**a**,**b**) Fire extinguishing sensing at a certain distance from the fire source by the multiparameter sensor; (**c**,**d**) ammeter and voltmeter readings of the sensor 10 cm away from the fire extinguishing point; (**e**,**f**) ammeter and voltmeter readings of the sensor 30 cm away from the fire extinguishing point.

In order to simulate the scene when firefighters extinguish the fire, water was used to extinguish the fire and the multiparameter sensor was used to monitor the change in temperature and humidity around the fire extinguishing point (Figure 8a,b). The voltmeter and ammeter readings from the multiparameter sensor are shown in Figure 8c,d. It can be inferred that the temperature and humidity at 10 cm away from the fire extinguishing point have changed, compared with 10 cm away from the fire source. According to Equation (4), the humidity at this time is 50%, and according to Equation (5), the temperature at this time is 55 °C. As shown in Figure 8e,f, the self-powered multiparameter sensor was used to monitor the fire extinguishing point at a distance of 30 cm. According to Equation (4), the humidity at this time is 50%, and according to Equation (5), the temperature at this time is 33 °C.

## 4. Conclusions

In summary, the ultrasensitive and self-powered multiparameter pressure–temperature–humidity sensor has been fabricated using ultra-flexible conductive silica aerogel. The ultra-flexible conductive silica aerogel was prepared by introducing PEDOT:PSS on a base of ultra-flexible silica aerogel, which was prepared by a diene synthesis reaction at atmospheric pressure. The piezoresistive effect of conductive silica aerogel, the electronic Seebeck effect, and the ion Seebeck effect of PEDOT:PSS have been applied to realize multiparameter sensing. More importantly, the sensor can be self-powered to realize the multiparameter sensing of pressure, temperature, and humidity which are converted into electrical signals, respectively. Compared with the traditional pressure sensor, the ultra-flexible silica aerogel has greatly increased sensitivity. The pressure sensitivity is as high as 54.88 kPa^−1^, and the detection limit is as low as 5 Pa. The temperature resolution is up to 0.1 k, and the response time to humidity is within 4 s. Multiparameter sensors have great potential due to their high efficiency and large-scale preparation. They are expected to be able to monitor human activities and fire-affected areas.

## 5. Materials and Methods

### 5.1. Materials

Vinyl trimethoxysilane (VTMS, 98%) and cetyltrimethylammonium chloride (CTAC) were purchased from Shanghai Aladdin Biochemical Technology Co., Ltd., Shanghai, China. Vinyldimethyldimethoxysilane (VMDMS, 98%), isoprene, and ZnCl2 (98%) were obtained from Shanghai Macklin Biochemical Co., Ltd., Shanghai, China. Ethyl alcohol, isopropanol, urea, acetic acid, and n-hexane were obtained from Shanghai Hutian Chemical Co., Ltd., Shanghai, China. PEDOT:PSS was received from Guangzhou aibang synthetic materials Co., Ltd., Guangzhou, China.

### 5.2. Sample Preparation

VTMS and VMDMS were used as precursor systems, combined with a diene synthesis reaction to prepare an ultra-flexible aerogel. The aerogel could be obtained in five simple steps. Firstly, a 15 mL acetic acid solution with a concentration of 5 mM was prepared in a glass beaker. Then, 0.8 g CTAC and 5 g urea were mixed in an acetic acid solution. An amount of 2.5 mL VTMS and 2.5 mL VMDMS were added into the mixed solution at the molar ratio of 1:1, and the solution was mixed and stirred for 30 min at room temperature for acid-catalyzed hydrolysis of alkoxysilanes. The second step was transferring the resulting transparent sol to an oven for gelation and aging at 80 °C over several hours. Urea hydrolysis to ammonia forms alkaline conditions to promote the polycondensation process. The third step was washing several times with isopropanol and n-hexane every 8 h. The fourth step was drying under ambient conditions. The fifth step was immersing the vinyl aerogel in an isoprene solution for 24 h, adding zinc chloride as a catalyst. The prepared aerogel was obtained after drying at atmospheric pressure (Figure S1).

**Figure 9:**
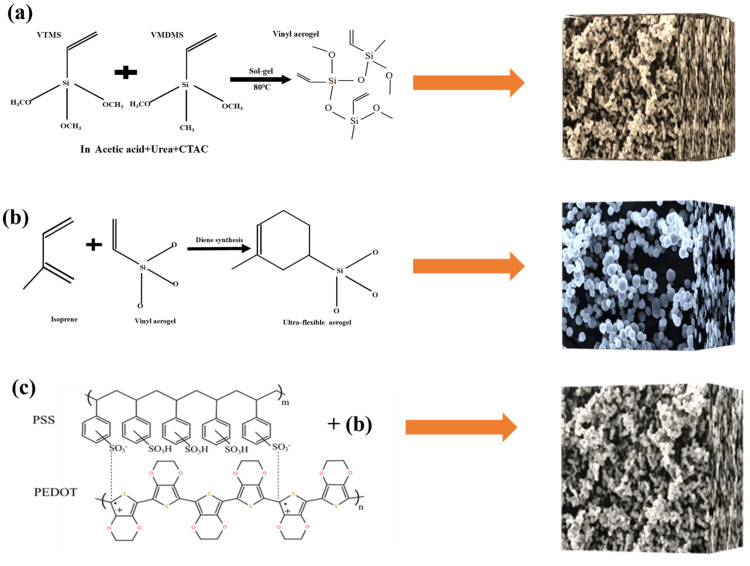
(**a**) Synthesis of the vinyl aerogel; (**b**) synthesis of the ultra-flexible aerogel; (**c**) preparation of conductive silica aerogel by compounding PEDOT:PSS.

**Figure 10:**
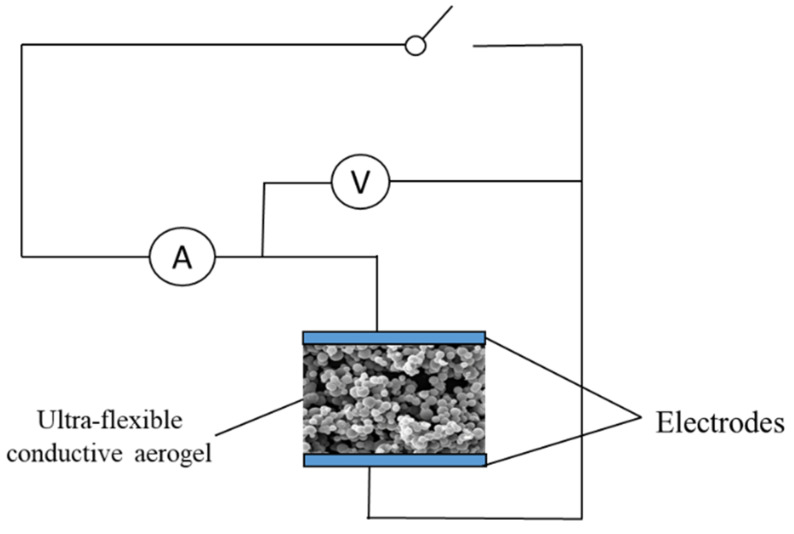
Schematic diagram of a multiparameter sensor setup.

The prepared aerogel was immersed in PEDOT:PSS (containing 5 vol% ethyl alcohol) and dried at 60 °C for 20 min. Thereupon, the PEDOT:PSS was evenly applied to the porous structure of ultra-flexible silica aerogel to prepare conductive silica aerogel (Figure 9). Clamping the ultra-flexible conductive silica aerogel between two copper electrodes, the prepared materials were assembled into a multiparameter sensor (Figure 10). Both sides of the copper electrode were bonded with conductive tape to ensure the continuous output of electrical signals.

### 5.3. Characterization of Samples

The microstructure and elemental analysis were observed under a scanning electron microscope-energy dispersive X-ray spectroscope (SEM-EDX) (TESCAN MIRA LMS, Czech). Before observation, the samples were put in a vacuum to spray platinum on their surface for adding conductivity. The thermal stability of all samples was reflected by thermogravimetric analysis (TG). It was measured by an instrument (TGA55, USA) at a heating rate of 10 °C·min^−1^ while continuously supplying nitrogen at a rate of 100 mL·min^−1^ [48]. The mechanical properties of the sample were measured by an electronic universal testing machine (Instron 5967, Instron, USA). For uniaxial compression tests, the 50 N weighing sensor was used to compress and decompress the aerogel at the rate of 3 mm/min (the diameter of samples was 12 mm and the height was 10 mm).

## Figures and Tables

**Table 1:** The element content comparison of aerogel without PEDOT:PSS and aerogel with PEDOT:PSS.

Samples	Si	C	O	S
Aerogel without PEDOT:PSS	30.59	46.20	23.00	0.00
Aerogel with PEDOT:PSS	24.21	53.28	21.27	0.81

**Table 2:** Comparison of pressure sensitivity with previously reported pressure sensors (PDMS: polydimethylsiloxane; OFET: organic field effect transistor; PVDF: polyvinylidene difluoride).

Type of Sensor	Materials	Sensitivity	Limit of Detection	Reference
Piezoresistivity	VMDM, VTMS/PEDOT:PSS	54.88 kPa^−1^	5 Pa	This work
Piezoresistivity	PDMS/PEDOT:PSS/PUD	10.32 kPa^−1^	23 Pa	[23]
Piezoelectricity/OFET	P (VDF-TrFE)	-	200 kPa	[24]
Piezoresistivity	Graphene	-	100 Pa	[25]
Piezoelectricity	Titanate/P (VDF-TrFE)	7 × 10^−4^ kPa^−1^	200 kPa	[26]
Piezoelectricity	PVDF	2 V kPa^−1^	1 kPa	[27]
Piezoresistivity	Tissue/Gold nanowires	1.14 kPa^−1^	13 Pa	[28]
Capacitance	Alumina ceramic	0.0035 kPa^−1^	100 kPa	[29]
Capacitance/OFET	PS-b-P2VP	1.76 kPa^−1^	17 Pa	[30]
Optical waveguide	PDMS	0.2 kPa^−1^	<1 kPa	[31]
Capacitance/OFET	PDMS/Rubrene	0.55 kPa^−1^	3 Pa	[32]

**Table 3:** Comparison of sensing performance with recently reported humidity sensors (MWCNT: multiwalled carbon nanotube; CNF: cellulose nanofiber; RGO: reduced graphene oxide; PDDA: poly(diallylimethyammonium chloride); PI: polyimide).

Materials	Fabrication	Output Signal	Response Time	Reference
VMDMS, VTMS/PEDOT:PSS	Soakage	current	4 s	This work
MWCNTs on paper	Drawing	current	470 s	[41]
CNF/MWCNTs	vacuum filtration	current	330 s	[42]
RGO/PDDA	layer-by-layer self-assembly	resistive	108 s	[43]
Paper	tape-attached	current	472 s	[44]
CNF/MWCNTs	vacuum filtration	current	333 s	[45]
MoS2	CVD	resistive	10 s	[46]
CNT/PI	in situ polymerization	resistive	5 s	[47]

## Data Availability

Not applicable.

## Supplementary Materials

The following supporting information can be downloaded at: https://www.mdpi.com/article/10.3390/gels9020162/s1, Figure S1: Macroscopic appearance of the silica aerogels without (a) and with (b) PEDOT: PSS; Figure S2: Scanning electron microscopies of silica aerogels with PEDOT: PSS and elemental mapping of silica aerogels with PEDOT: PSS; Figure S3: Compressive stress−strain curves for ultra-flexible conductive silica aerogels; Figure S4: Pore size distribution of sample; Figure S5: The water contact angle of sample; Figure S6: The schematic diagram of the specific fire scene; Video S1: Resistance changes with pressure.
