# Supplementary material for: Ultrasensitive and Self-Powered Multiparameter Pressure–Temperature–Humidity Sensor Based on Ultra-Flexible Conductive Silica Aerogel
Source: Gels. 2023 Feb 17;9(2):162. doi: 10.3390/gels9020162 (PMC9956380; doi:10.3390/gels9020162)
Supplement: Supplementary file 1 [file gels-09-00162-s001.zip › gels-2201509 - supplementary.pdf]

Ultrasensitive and Self-Powered Multiparameter Pressure–Temperature–Humidity Sensor Based on Ultra-Flexible Conductive Silica Aerogel

Song He [1], Chunhua Du [1], Hongliang Sheng [2,*], Chunxiang He [1], Xinyu Liu [1], Xin Jin [1], Qilin Chen [1] and Fuliang Tian [1]

[1] School of Safety Science and Emergency Management, Wuhan University of Technology, Luoshi Road 122, Wuhan 430070, China
[2] Wuhan Building Material Industry Design & Research Institute Co., Ltd., Wuhan 430200, China
* Correspondence: sheng.hl@sinoma-wbmdi.cn

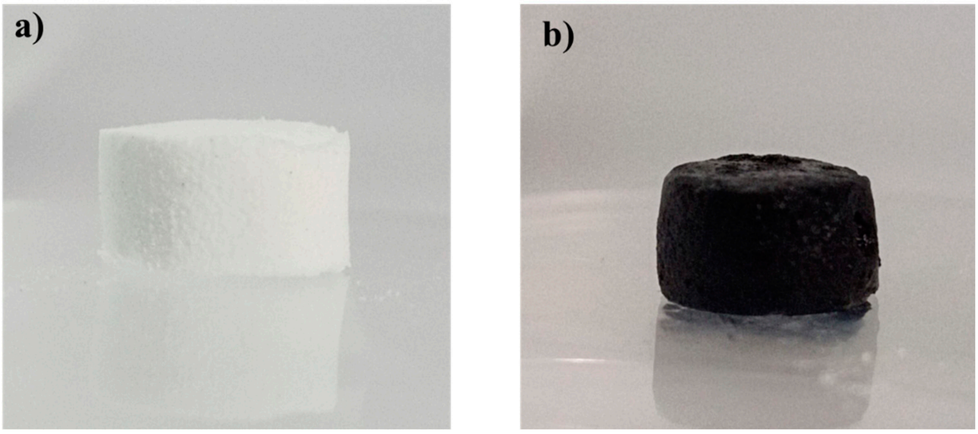

Figure S1. Macroscopic appearance of the silica aerogels without (a) and with (b) PEDOT: PSS.

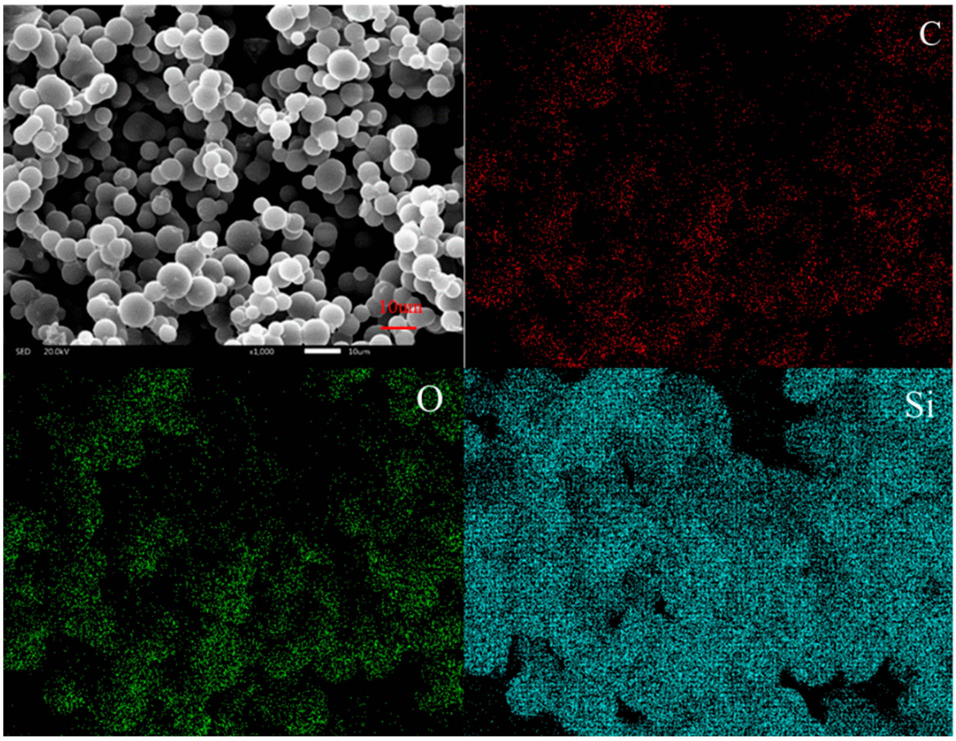

Figure S2. Scanning electron microscopies of silica aerogels with PEDOT: PSS and elemental mapping of silica aerogels with PEDOT: PSS.

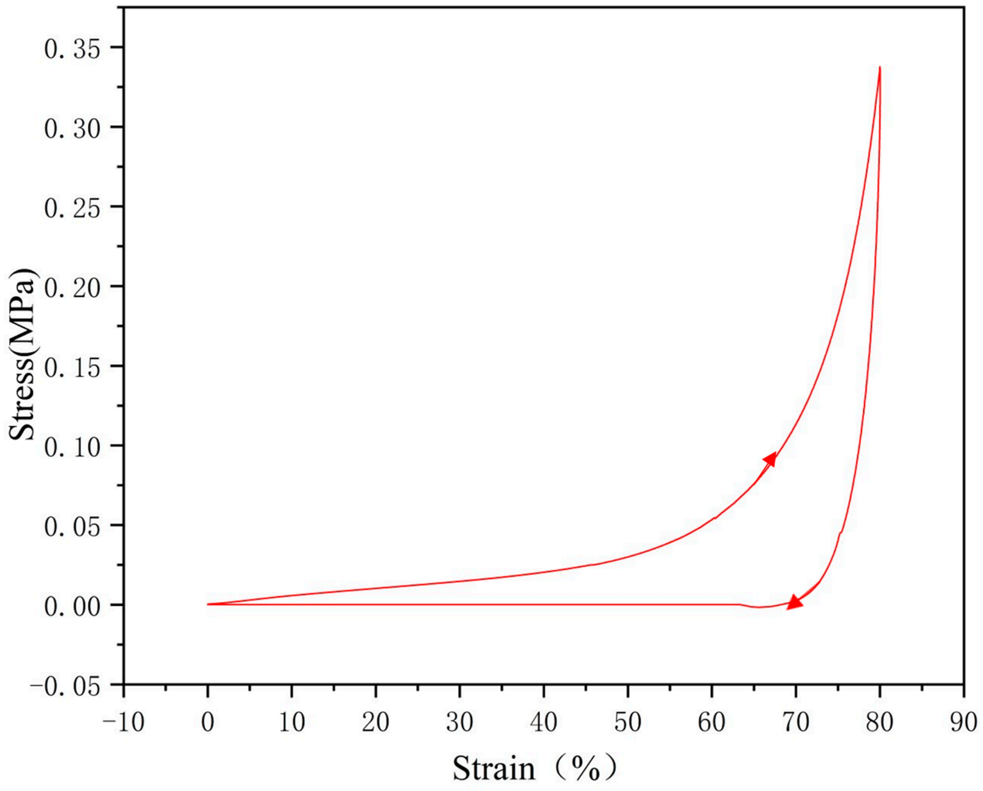

Figure S3. Compressive stress−strain curves for ultra-flexible conductive silica aerogels.

In order to evaluate the mechanical properties of materials, Young's modulus is proposed. Young's modulus is a term in material mechanics. Elastic materials will produce positive strain when they bear positive stress, which is defined as the ratio of positive stress to positive strain. The formula [1] is recorded as Equation (S1)

$$E=\sigma/\varepsilon \quad \text{(S1)}$$

where E is Young's modulus, σ indicates positive stress, ε indicates positive strain. The figure of Young's modulus is smaller, the flexibility of the material is greater when the samples is compressed or stretched. According to Figure S3, the Young's modulus of the sample is as low as 0.05 Mpa. The low elastic modulus gave the aerogel a low detection limit, which provides an excellent piezoresistive effect for multi parameter sensors and improves the sensitivity of pressure sensing.

From the scanning electron microscope of the sample, it can be seen that the pore size of sample is micron level. But the automatic specific surface area tester by nitrogen adsorption method is based on BJH algorithm, which has restrictions on the measurement of apertures. The nitrogen adsorption method is applicable to the test of nanoscale materials. Therefore, BJH algorithm could not reflect the pore distribution observed from SEM. Similarly, other parameters measured by nitrogen adsorption method, such as pore volume, specific surface area and average pore diameter cannot correctly characterize the prepared aerogels. Nitrogen adsorption method can only measure the specific surface area with pore diameter below 220 nm. But the samples are basically macroporous structure. Therefore, it is not suitable to use BJH algorithm to measure and characterize the pore size distribution of the prepared aerogels.

According to the investigation, the pore size distribution of macroporous aerogels can be measured by mercury intrusion method. However, this method requires a certain stiffness of the samples to press mercury into the holes of the tested samples. The pore size distribution curve of sample by mercury intrusion method is shown in Fig.S4. It can be clearly seen that the pores in the samples are basically macroporous structure, with the

largest number of pores of about 1.6 um. The pore size distribution obtained by mercury intrusion method is consistent with that observed by scanning electron microscope. Mercury intrusion method is also called mercury porosity method. It is a method to determine the pore size distribution of some mesopores and macropores. Therefore, according to the principle of mercury intrusion method, the data of pore size distribution of the prepared samples can be obtained. The data of nitrogen adsorption desorption curve, specific surface area and pore volume cannot be accurately obtained by mercury intrusion method.

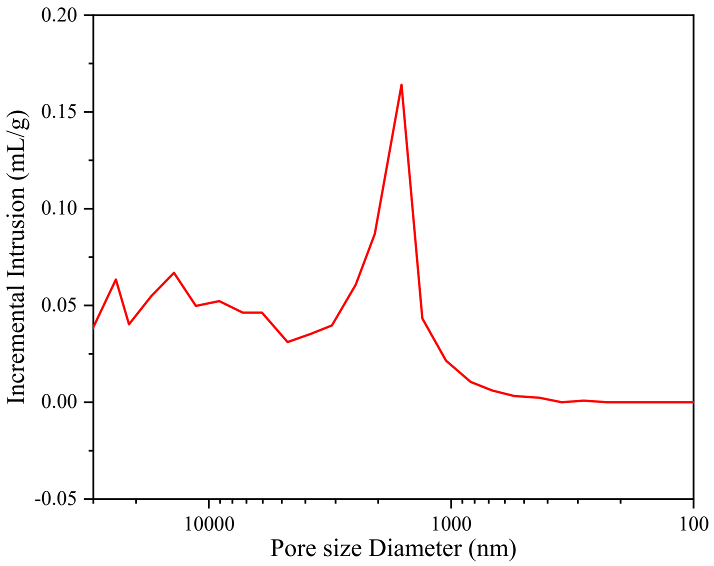

Figure S4. Pore size distribution of sample.

The water contact angle of sample is shown in the Figure S5. The water contact angle reaches about 118°. The water contact angle will demonstrate the long-term stability of the material, as hydrophilic silica is known to be destroyed by contact with humidity.

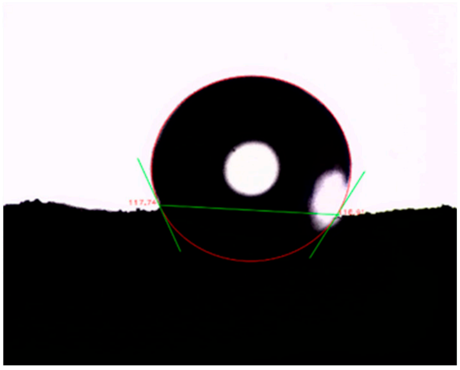

Figure S5. The water contact angle of sample.

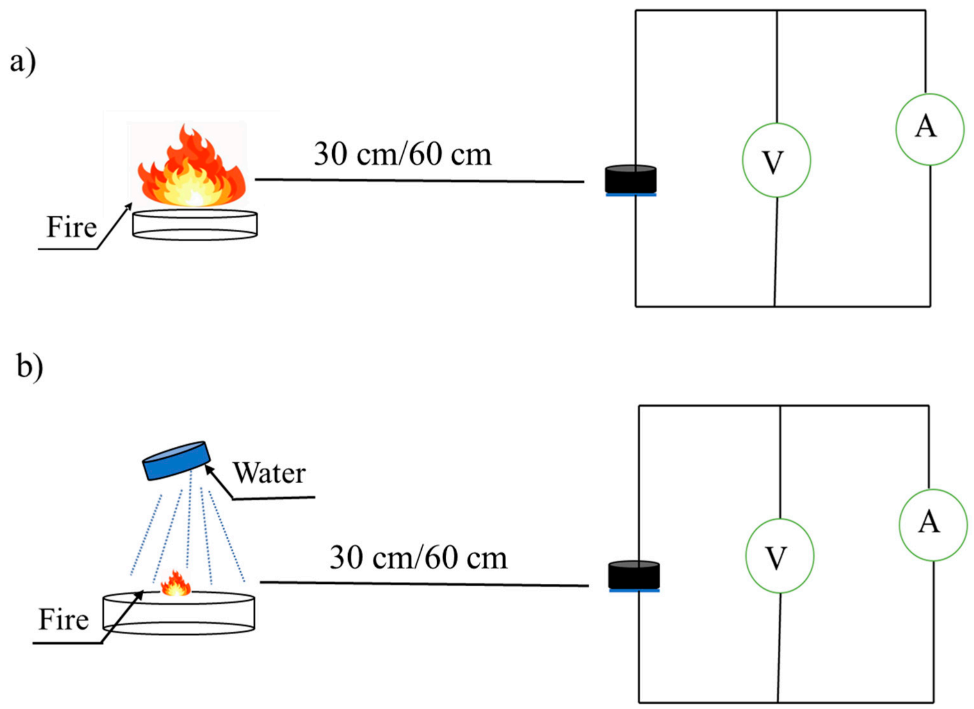

Figure S6. The schematic diagram of the specific fire scene.

Notes and References

1. A.V. Rao, S.D. Bhagat, H. Hirashima, Synthesis of flexible silica aerogels using methyltrimethoxysilane (MTMS) precursor, Journal of Colloid & Interface Science 300 (2006) 279-285.
